# Which health technologies should be funded? A prioritization framework based explicitly on value for money

**DOI:** 10.1186/2045-4015-1-44

**Published:** 2012-11-26

**Authors:** Ofra Golan, Paul Hansen

**Affiliations:** 1 Unit for Genetic Policy & Bioethics, The Gertner Institute for Epidemiology & Health Policy Research, Tel Hashomer, Israel; 2 Department of Economics, University of Otago, Dunedin, New Zealand

**Keywords:** Health technology, Prioritization, Basket committee, Multi-criteria decision analysis, Points system, Value for money

## Abstract

**Background:**

Deciding which health technologies to fund involves confronting some of the most difficult choices in medicine. As for other countries, the Israeli health system is faced each year with having to make these difficult decisions. The Public National Advisory Committee, known as ‘the Basket Committee’, selects new technologies for the basic list of health care that all Israelis are entitled to access, known as the ‘health basket’. We introduce a framework for health technology prioritization based explicitly on value for money that enables the main variables considered by decision-makers to be explicitly included. Although the framework’s exposition is in terms of the Basket Committee selecting new technologies for Israel’s health basket, we believe that the framework would also work well for other countries.

**Methods:**

Our proposed prioritization framework involves comparing four main variables for each technology: 1. Incremental benefits, including ‘equity benefits’, to Israel’s population; 2. Incremental total cost to Israel’s health system; 3. Quality of evidence; and 4. Any additional ‘X-factors’ not elsewhere included, such as strategic or legal factors, etc. Applying methodology from multi-criteria decision analysis, the multiple dimensions comprising the first variable are aggregated via a points system.

**Results:**

The four variables are combined for each technology and compared across the technologies in the ‘Value for Money (VfM) Chart’. The VfM Chart can be used to identify technologies that are good value for money, and, given a budget constraint, to select technologies that should be funded. This is demonstrated using 18 illustrative technologies.

**Conclusions:**

The VfM Chart is an intuitively appealing decision-support tool for helping decision-makers to focus on the inherent tradeoffs involved in health technology prioritization. Such deliberations can be performed in a systematic and transparent fashion that can also be easily communicated to stakeholders, including the general public. Possible future research includes pilot-testing the VfM Chart using real-world data. Ideally, this would involve working with the Basket Committee. Likewise, the framework could be tested and applied by health technology prioritization agencies in other countries.

## Background

No health system in the world has sufficient resources to be able to afford all available health care technologies – i.e. pharmaceuticals, medical procedures, equipment, devices and health services. Inevitably, therefore, technologies must be prioritized. Deciding which technologies to fund (and which not to) involves confronting some of the most difficult choices in medicine.

As for other countries, the Israeli health system, which is committed to an explicit prioritization process for new technologies, is faced each year with having to make these difficult decisions. The National Health Insurance Law determines a basic list of health care that all Israelis are entitled to access, known as the ‘health basket’ [1]. New technologies are added to the health basket once a year depending on the funds available, which is just a small fraction of the total amount requested for new technologies. For example, in 2010 the Committee had to decide how to allocate a budget of 300 million shekels (approximately US$85 million) across 430 candidate technologies – mostly pharmaceuticals, and with a combined total cost of more than a billion shekels – resulting in 61 being added to the health basket [2].

The Israeli mechanism for updating the health basket comprises two main elements. First, health technology assessments are performed by the Health Technologies Forum at the Ministry of Health. The added value of each technology submitted for addition to the basket is assessed with respect to its clinical, epidemiological and economic characteristics, including its predicted impact on the available budget. Second, and as discussed in greater detail in the Discussion section below, informed by these assessments, the Public National Advisory Committee, known as ‘the Basket Committee’, selects new technologies for the basket based on the application of pre-defined criteria related to the technologies’ costs and benefits as well as ethical and legal considerations [3,4]. This prioritization process is considered by many health policy analysts, both in Israel and abroad, to be internationally ground-breaking [1,5-7]. It is unique with respect to its comparison of all proposed technologies together (numbering in the hundreds, as in the example above) subject to a budget constraint, and for its integration of professionally-performed technology assessments with pre-defined criteria and ethical and legal considerations [5].

The criteria used by the Basket Committee [8] are mostly universal, as we established in an earlier study [9] in which we surveyed the literature (using PubMed and Google) to discover the main criteria and other considerations for prioritizing new technologies in use internationally. Encompassing 11 countries and the US state of Oregon, we were able to distinguish three main groups of criteria: need, appropriateness and clinical benefits; efficiency (including cost-effectiveness); and equality, solidarity and other ethical or social values. As well, the quality of clinical evidence and factors related to strategic issues and procedural justice were explicitly considered in several countries.

Although these criteria and other considerations are qualitatively similar across countries, their relative importance is different, reflecting international differences in how the inevitable conflicts and trade-offs between competing moral principles are dealt with. The Basket Committee is mandated to resolve such conflicts and trade-offs between competing moral principles based on the value judgments of its members. According to the Ministry of Health, “The criteria which guide the Committee’s work *are not* hierarchical and *not* equivalent in their importance. The criteria should be used as a qualitative (and not quantitative) guideline for the Committee’s decisions” (p. 8) [8]. However, no guidance is offered about how to weight and balance the criteria and other considerations relative to each other [10].

“For a set of moral considerations to be useful to decision makers, some guidance on weighting of different considerations needs to be given.” (p. 57) [11]. This requirement, or, conversely, the absence of such guidance for decision-makers internationally, has been pointed out many times by researchers [10,12-16]. Stafinski et al. [16] assembled a comprehensive inventory of decision-making processes used in 20 countries (not including Israel). “While information requirements of all processes appeared substantial and decision-making factors comprehensive, the way in which they were utilized was often unclear, as were approaches used to incorporate social values or equity arguments into decisions.” (p. 476). Although cost-effectiveness evidence is the main consideration for prioritization agencies such as, for example, the UK’s National Institute for Health and Clinical Excellence, other factors are also taken into account [17]; however, it is not clear how such factors are incorporated in practice [15]. The advisability of there being more structure in the decision-making processes employed by the Israeli Basket Committee is suggested by a forthcoming review of the Committee’s decisions and the reported comments of its members [18].

Informed by our earlier study’s results [9], this article introduces a framework for health technology prioritization based explicitly on value for money that enables the main variables considered by decision-makers such as the Basket Committee to be explicitly included. The main challenge addressed by what we refer to as the ‘Value for Money Chart’ is how to combine these variables in a transparent and intuitively appealing way that helps decision-makers to focus on the inherent tradeoffs when choosing new technologies subject to a budget constraint. Although our exposition of the framework is couched in terms of the Basket Committee selecting new technologies for Israel’s health basket, we believe that the framework would also work well for other countries as well as for other levels of health technology prioritization (e.g. regional or service providers).

## Methods

Informed by the results of our earlier study [9] mentioned above, our proposed framework involves comparing four main variables for each technology (from a societal perspective): 1. Incremental benefits, including ‘equity benefits’, to Israel’s population; 2. Incremental total cost to Israel’s health system; 3. Quality of evidence; and 4. Any additional ‘X-factors’ not elsewhere included, such as strategic or legal factors, etc. It is important to appreciate that these first two variables are at the aggregate level for each technology – i.e. in terms of the effects of the overall intervention involving the technology on Israel’s population and health system respectively – rather than at a disaggregated level (e.g. per patient treated). Each of the four variables is now explained in turn.

### Incremental benefits, including ‘equity benefits’, to Israel’s population

Health technologies have three fundamental purposes: to save lives, to prolong lives, and to improve (or preserve) health-related quality-of-life (HRQoL); in addition, health resources are used to reduce health inequalities [11]. Thus, the ‘incremental benefits, including equity benefits, to Israel’s population’ potentially available from each technology has four main dimensions: life-saving, life-prolongation, HRQoL improvements, and ‘equity benefits’.

‘Equity benefits’ relates to the various aspects of equity that ought to be taken into account when assessing health technologies [19]. Consistent with our earlier study [9], two main aspects (sub-dimensions) are included in our proposed framework: the extent to which, if the technology were not to be funded, patients would be denied treatment due to a lack of alternative treatments or difficulties accessing them; and the existence of other important equity-related social or ethical benefits, such as the technology being targeted at specific populations with *prima facie* special claims (e.g. children or minorities) or serving to reduce health gaps (inequalities), etc.

It should be clear from the discussion above that this first variable in the framework, ‘incremental benefits, including equity benefits, to Israel’s population’, is multi-dimensional. In order to be able to compare this variable against the framework’s three other variables (explained below) – for which, as discussed below, uni-dimensional measures exist – some means of aggregating the dimensions in a way that reflects their relative importance and quantifies tradeoffs so that a uniform measure (index) of incremental benefits can be created is required.

An obvious way of aggregating the three dimensions of life-saving, life-prolongation and HRQoL improvements is to use Quality-Adjusted Life Years (QALYs). However, for *new* health technologies QALY data may not be available; and this ‘data problem’ is magnified if, as is the case for Israel’s Health Basket, there are many technologies to be evaluated at once. A practical alternative to using QALYs is to focus directly on the underlying dimensions themselves (albeit their data may be less than perfect too), while also recognizing that each dimension comprises various sub-dimensions. Thus, the life-prolongation dimension incorporates increases in life expectancy and the HRQoL at which the additional life years are experienced; the HRQoL-improvements dimension incorporates the magnitude of the HRQoL gains, their duration, and baseline HRQoL (‘need’).

Whichever of these two possible approaches is used, some means of aggregating either QALYs gained with ‘equity benefits’ (itself multi-dimensional) or the underlying dimensions is required. A common methodology from the field of multi-criteria decision analysis is to use a points system (sometimes also referred to as a ‘scoring’, ‘linear’ or ‘point-count’ system).

A points system is a schedule of ‘point values’ (or ‘weights’) representing both the relative importance of the dimensions and their degree of achievement; an example for ranking new technologies that we developed in our earlier study [9] appears in Table 1, where the point values were derived from a convenience sample of respondents (discussed later below) and are reported here for illustrative purposes only. Points systems, which have been found to be accurate in thousands of ‘health’ and ‘non-health’ applications [20], are widely used for diagnostic and treatment-based decision-making [21]. Other health applications include prioritizing patients within specific elective services in the UK, New Zealand and Canada [22] and allocating transplant organs by the United Network of Organ Sharing [23]. In the present context, using a points system involves rating each technology according to its performance on each dimension and then summing the corresponding point values to get a ‘total score’ by which the technologies are ranked. Later in this section we explain how to derive point values.

**Table 1:** Illustrative points system for the incremental-benefits variable

**Dimensions**	**Points (weights)**
**Lives saved, including ‘statistical’ lives (i.e. cure or reduced risk of death)**	
None (or not yet known)	0
Few: 1-50 lives saved	0.091
Some: 51-250 lives saved	0.192
Many: 251-500 lives saved	0.268
Very many: > 500 lives saved	**0.343**
**Life-prolongation benefits – in terms of increase in life expectancy and its quality-of-life, and number of patients affected**	
None/Very small (or not yet known)	0
Small benefits	0.053
Medium benefits	0.152
Large benefits	**0.244**
**Quality-of-life gains – in terms of baseline QoL, size of QoL gains and duration, and number of patients affected**	
None/Very small (or not yet known)	0
Small QoL gains	0.051
Medium QoL gains	0.138
Large QoL gains	**0.217**
**If this technology were *not *to be funded …**	
Many/most patients **will** be able to pay for it themselves (privately)	0
Many/most patients **will get an alternative** treatment (less effective) already funded by government	0.055
Many/most patients will **not** receive any treatment for condition	**0.108**
**Other important social or ethical benefits, e.g. targeted to children/minorities; reduces health gaps, etc**	
None/Very small (or not yet known)	0
Yes	**0.087**

*Note*: The bolded values represent the relative weights of the dimensions overall (i.e. the bolded values sum to unity).

### Incremental total cost to Israel’s health system

A technology’s ‘incremental total cost to Israel’s health system’ (i.e. as explained earlier, at the aggregate level of the overall intervention involving the technology) can be measured in net present value (NPV) terms. This NPV includes all expected future spending, net of any cost savings to the health system, over the intervention’s lifetime – i.e. the same lifetime over which the incremental benefits referred to above are recognized. Alternatively – as in our illustration in the next section – both the costs and benefits could be in per annum terms. All else being equal, a technology’s incremental total cost to Israel’s health system will be positively related to the intervention’s time horizon and also to the number of patients to be treated, which depends on how eligibility is defined – which in turn determines the incremental benefits possible from the technology.

### Quality of evidence

Especially for *new* technologies, there are likely to be significant differences between technologies with respect to the quality of their clinical evidence. For example, if two technologies are assessed as having the same incremental benefits (the first variable above), but one assessment is based on higher quality evidence than the other, then they ought to be differentiated in this regard – so that the technology with the higher quality evidence receives higher priority (all else being equal).

Several grading schemes for assessing quality of evidence [24-26] are potentially available, of which the GRADE system [24] is perhaps the best known. GRADE, which defines quality of evidence as “the extent to which we can be confident that an estimate of effect is correct” (p. 1490), incorporates four key elements: study design, study quality, consistency (the similarity of effect estimates across studies), and directness (the extent to which the people, interventions and outcome measures in the studies are similar to those of interest). In general, however, caution should be exercised when applying grading schemes. “It should be noted that not all the schemes take into account the generalizability of the findings of the review to routine clinical practice. This should always be a consideration when drawing up the implications or if making recommendations.” (p. 82) [27].

### Any additional ‘X-factors’ not elsewhere included, such as strategic or legal factors, etc

This last variable in the prioritization framework is, in effect, a catchall for any residual special circumstances of *a priori* uncertain importance that ought to be recognized. This variable is meant to be implemented simply as a ‘flag’ to alert decision-makers that such additional X-factors – which could be positive or negative (i.e. supportive of the technology being added to the health basket, or not) – ought to be considered, on a technology-by-technology basis. For example, the technology of contraceptives for teenage girls involves unique religious, ethical and social considerations that most people would probably agree ought to be considered.^a^

There would be no need to recognize such X-factors if the first three variables discussed above (incremental benefits, incremental costs, quality of evidence) perfectly captured all relevant considerations for prioritizing technologies. In practice, though, this is unlikely, as there will almost always be particular technologies for which there are additional factors that ought to be considered. The key point is that if decision-makers think that a given technology’s X-factors should, in effect, over-ride its performance on the three other variables with respect to being selected or rejected for the basket then the reasons for doing so should be made explicit.

### Creating a points system for the incremental-benefits variable

As discussed earlier, ‘incremental benefits, including equity benefits, to Israel’s population’ comprises multiple dimensions that are aggregatable into a single measure using a points system. An example of a points system developed in our earlier study [9] appears in Table 1. The reported point values, which were derived from a convenience sample recruited through the first author’s professional networks,^b^ are applied for illustrative purposes in the next section. Were the framework being used by the Basket Committee, the points system’s dimensions and their ‘performance’ levels would likely need to be refined; likewise, the point values – the determination of which is explained next – would reflect the Committee’s preferences.

Thus, after a points system’s dimensions and levels have been specified, their point values, reflecting the relative importance of the dimensions to decision-makers, need to be determined. Several methods and software for implementing them are available, as surveyed in [15,28] and [29] respectively. Methods that involve decision-makers expressing a *choice* between the alternatives of interest, such as conjoint analysis (also known as ‘discrete choice experiments’ [30]) which has been recommended as the best overall approach for valuing health benefits [31], are generally favored. “The advantage of choice-based methods is that choosing … is a natural human task at which we all have considerable experience, and furthermore it is observable and verifiable.” (p. 145) [32].

An example of a choice-based methodology is the PAPRIKA method [33]. PAPRIKA, which is an acronym for ‘**P**otentially **A**ll **P**airwise **R**an**K**ings of all possible **A**lternatives’, and software for implementing the method known as ‘1000Minds’ [34] were co-invented by the second author (from whom or via [34] the software is available for free to unfunded academic users). PAPRIKA and 1000Minds were used in our earlier study [9], and also here in our prioritization framework. Other applications of the method and software include prioritizing patients for elective surgery [33,35,36], referring patients for rheumatology services [37], classifying individuals by their risks of developing rheumatoid arthritis [38], and measuring patients’ responses in clinical trials for chronic gout [39].

**Figure 1:**
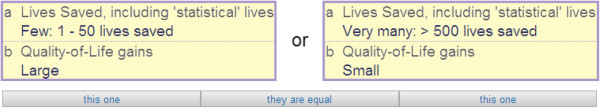
Example of a pairwise-ranking question for determining point values.

The PAPRIKA method involves decision-makers – Basket Committee members and/or their constituencies^c^ if the Committee were to use the framework – using their judgments to pairwise rank a series of hypothetical technologies with respect to their relative priority for addition to the health basket. The pairs of hypothetical technologies, which are presented in random order, are defined on two dimensions at-a-time so that decision-makers are forced to confront a trade-off between the dimensions with respect to their relative importance for prioritizing technologies. An example of a pairwise-ranking question (a screen from the 1000Minds software) appears in Figure 1.

Although it is possible for decision-makers to answer the questions individually (with their results ‘averaged’), based on the second author’s experience in similar applications [36], we believe it is better to have decision-makers answer the questions as a group by having them vote on each question and discuss any significant disagreements and reach consensus (not necessarily unanimity).

The PAPRIKA method ensures that the number of questions that decision-makers have to answer is minimized by, each time a question is answered, eliminating all other possible questions that are implicitly answered as corollaries of those already answered. This is achieved via the method’s application of the ‘transitivity’ property; for example, if decision-makers rank hypothetical technology ‘A’ ahead of technology ‘B’ and also ‘B’ ahead of technology ‘C’, then, logically (by transitivity), ‘A’ must be ranked ahead of ‘C’ (and so the 1000Minds software would not ask a question pertaining to this third pairwise ranking). The number of questions answered by decision-makers depends on the number of dimensions and levels in the points system. The points system in Table 1, for example, requires decision-makers to answer about 40 questions involving trade-offs between two dimensions at-a-time. Based on the answers, the 1000Minds software uses mathematical methods (explained in detail in [33]) to calculate the point values (reflecting the relative importance of the dimensions to decision-makers).

After each technology has been rated on the points system’s dimensions, the corresponding point values are summed to get a ‘total score’ for each technology. Equipped thus with a single value for the incremental-benefits variable for each technology, plus a value for each of the three other variables (compiled separately) included in the framework, the four variables are ready to be combined in what we refer to as the ‘Value for Money Chart’. This is presented in the next section.

## Results

**Figure 2:**
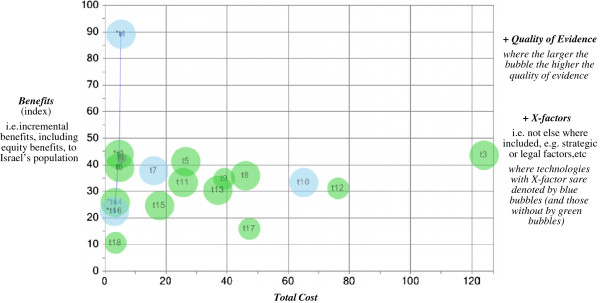
**Value for money chart, with 18 illustrative technologies (see Table**2**for their names).**

The four variables discussed above for each technology can be displayed in the Value for Money (VfM) Chart, as illustrated in Figure 2. Although the VfM Chart is capable of representing potentially any number of technologies (limited only by the chart’s size), for simplicity and so that the chart can be easily read on a journal page, we have restricted ourselves to 18 illustrative technologies. The technologies’ names corresponding to their labels in Figure 2 and their underlying data, including their ratings on the illustrative points system (Table 1), are reported in Table 2.

**Table 2:** Data for the 18 illustrative technologies in Figures 2 and 3

**Technology (indication, number of potential patients)**	**Lives Saved**	**Life-Prolongation Benefits**	**Quality-of-Life (QoL) gains**	**If this technology were*not*to be funded …**	**Other important social or ethical benefits, etc**	**Total cost (millions of shekels, annual)**	**Quality of evidence**	**X-factors**
t1. Smoking cessation drugs (smokers, 6000)	Very many: >500 lives saved	Large benefits	Large QoL gains	Many/most patients will be able to pay for it themselves (privately)	Yes	5.29	high	not smoking is a personal choice
t2. Taxotere (head and neck cancer, 200)	None (or not yet known)	Large benefits	None/very small (or not yet known)	Many/most patients will not receive any treatment for condition	Yes	4.6	high	none
t3. Herceptin (breast cancer – adjuvant treatment, 700)	Few: 1-50 lives saved	Medium benefits	Small QoL gains	Many/most patients will get an alternative treatment (less effective) already funded by government	Yes	124	high	none
t4. Elaprase (Hunter syndrome, 3)	Few: 1-50 lives saved	None/very small (or not yet known)	Medium QoL gains	Many/most patients will not receive any treatment for condition	Yes	5.45	poor	orphan drug
t5. Visudyne (age-related macular degeneration, 1050)	None (or not yet known)	None/very small (or not yet known)	Large QoL gains	Many/most patients will not receive any treatment for condition	Yes	26.47	high	none
t6. Left-ventricular assist devices (terminal heart failure, 12)	Few: 1-50 lives saved	Small benefits	Small QoL gains	Many/most patients will not receive any treatment for condition	Yes	4.83	high	none
t7. Statins (hypercholesterolemia, 5600)	None (or not yet known)	Medium benefits	Medium QoL gains	Many/most patients will be able to pay for it themselves (privately)	Yes	16	high	strategic considerations
t8. Pain relief (neuropathic pain, 14,250)	None (or not yet known)	None/very small (or not yet known)	Large QoL gains	Many/most patients will get an alternative treatment (less effective) already funded by government	Yes	46	high	none
t9. Revlimid (multiple myeloma – 3^rd^-line treatment, 200)	None (or not yet known)	Medium benefits	None/very small (or not yet known)	Many/most patients will not receive any treatment for condition	Yes	39	medium	none
t10. Dental care (children, 20,000)	None (or not yet known)	None/very small (or not yet known)	Medium QoL gains	Many/most patients will not receive any treatment for condition	Yes	65	high	political considerations
t11. Growth hormone (short-statured children, 3900)	None (or not yet known)	None/very small (or not yet known)	Medium QoL gains	Many/most patients will not receive any treatment for condition	Yes	25.6	high	none
t12. Avastin [Bevacizumab] (colon cancer, 700)	None (or not yet known)	Medium benefits	Small QoL gains	Many/most patients will not receive any treatment for condition	None/Very small (or not yet known)	76.27	medium	none
t13. Over-active bladder drugs (urinary urge, incontinence, 21,000)	None (or not yet known)	None/very small (or not yet known)	Large QoL gains	Many/most patients will be able to pay for it themselves (privately)	Yes	37	high	none
t14. Fuzeon (HIV, 45)	None (or not yet known)	Medium benefits	Small QoL gains	Many/most patients will get an alternative treatment (less effective) already funded by government	None/Very small (or not yet known)	3.33	high	none
t15. Long-acting insulins (diabetes, 10,000)	None (or not yet known)	Small benefits	Medium QoL gains	Many/most patients will get an alternative treatment (less effective) already funded by government	None/Very small (or not yet known)	17.83	high	none
t16. Contraceptives (adolescent girls, 20,000)	None (or not yet known)	None/very small (or not yet known)	Medium QoL gains	Many/most patients will be able to pay for it themselves (privately)	Yes	3.11	high	socio-ethical, religious considerations
t17. Erbitux (colon cancer – for KRAS mutation negative, 210)	None (or not yet known)	Small benefits	Small QoL gains	Many/most patients will get an alternative treatment (less effective) already funded by government	None/Very small (or not yet known)	47.26	medium	none
t18. Humira (psoriatic arthritis, 60)	None (or not yet known)	None/very small (or not yet known)	Small QoL gains	Many/most patients will get an alternative treatment (less effective) already funded by government	None/Very small (or not yet known)	3.49	medium	none

Some of these technologies were chosen for inclusion here because of the Israeli public’s interest in them, and others because they represent a diverse range of characteristics. They are based on realistic data that were presented to or determined by the various Basket Committees mostly over the period 2005-8.^d^ Each technology’s ‘performance’ on each dimension, as well as the quality of evidence, were determined by the first author (OG) from her understanding of the data and, ultimately, her judgment. Accordingly, it should be recognized that the 18 technologies are presented solely for illustrative purposes and should not be regarded as the same as actual technologies discussed by the Basket Committee (various years); and therefore it is not appropriate nor feasible to compare the Committee’s decisions with the illustrative ones presented here.

As can be seen in Figure 2, the vertical axis of the VfM Chart displays each technology’s total score (as explained in the previous section), reflecting its ‘incremental benefits, including equity benefits, to Israel’s population’ – as produced here (for illustrative purposes) by applying the points system from our earlier study [9] (Table 1) to the technologies’ ratings (Table 2). The horizontal axis displays each technology’s ‘incremental total cost to Israel’s health system’. The size of the bubble used to represent each technology is in proportion to the ‘quality of evidence’. Finally, a blue bubble (or lighter shade if the chart is in black and white) indicates ‘any additional ‘X-factors’ not elsewhere included, such as strategic or legal factors, etc.’

### Which technologies are good value for money?

Decision-makers (e.g. the Basket Committee) should focus their attention first on the technologies in the VfM Chart’s top-left quadrant – with high *Benefits* and low *Total Cost* – while also being mindful of each technology’s *Quality of Evidence* and any *X-factors*. These technologies represent relatively good value for money. In contrast, technologies in the bottom-right quadrant – with low *Benefits* and high *Total Cost* – represent poor value for money.

**Figure 3:**
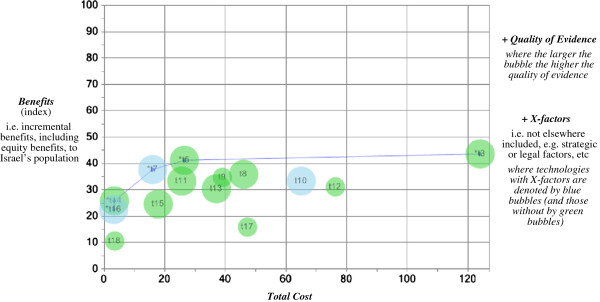
Value for money chart, after 4 technologies have been selected.

Possible acceptable tradeoffs between the *Benefits* and *Total Cost* variables on the chart’s axes are in a nor-easterly/sou-westerly direction, conditional on *Quality of Evidence* and *X-factors*. In other words, higher *Total Cost* can be compensated for by higher *Benefits*, all else (i.e. *Quality of Evidence* and *X-factors*) being equal. In this respect, ‘best value’ candidate technologies are identified by the (upward-sloping) frontier in the VfM Chart (see Figure 2 again and also Figure 3 later below). This frontier, known as the ‘Pareto (efficiency) frontier’, identifies ‘dominant’ technologies in the sense that, compared to them, no other technologies have *both* lower *Total Cost* and higher *Benefits*. (In contrast, the further away a technology is from the frontier in a sou-easterly direction, the lower is its value for money.)

The VfM Chart is useful for comparing technologies’ effectiveness, affordability and efficiency (i.e. cost-effectiveness). That is, all else being equal, the closer a technology is to the vertical axis, the more affordable it is in terms of having a lower *Total Cost*; the further away a technology is from the horizontal axis, the more effective it is in terms of having higher *Benefits*. The steeper is the ray that can be drawn from the chart’s origin to each technology, the more ‘efficient’ the technology is in terms of having a higher *Benefits / Total Cost* ratio.

### Which technologies should be funded?

Prioritizing technologies involves the Basket Committee mulling over alternative affordable combinations of the technologies represented in the VfM Chart to arrive at what the Committee considers to be the ‘optimal portfolio’ of technologies. In essence, the Committee should aim to maximize the aggregate *Benefits* from the technologies to be added to the health basket subject to the budget constraint and given the technologies’ *Total Cost*, *Quality of Evidence* and *X-factors*. This involves a process of trial-and-error.^e^ However, for some technologies it is likely to be relatively easy for the Committee to decide whether they should be added to the health basket or not, whereas other technologies will require more deliberation.

For example, with reference to the illustrative technologies displayed in the VfM Chart in Figure 2, it is easy to imagine that decision-makers would immediately select smoking cessation drugs (t1) and Taxotere (t2) (for the technologies’ names see Table 2). No other technologies are better (have higher *Benefits*) and these two are amongst the cheapest available (lowest *Total Costs*) – assuming *X-factors* for smoking cessation drugs do not militate against this technology’s selection. Conceivably, the Committee might choose Elaprase (t4), despite its poor *Quality of Evidence*, due to its high *Benefits* relative to *Total Cost* (arguably, this is consistent with the practice of Israeli Basket Committees of selecting life-saving orphan drugs).

The next three technologies with the highest *Benefits* are Herceptin (t3), Visudyne (t5) and left-ventricular assist devices (t6). However, Herceptin has only marginally higher *Benefits* than these two other technologies but is approximately 98 million and 119 million shekels more expensive respectively. Therefore, given these data, it would be understandable if the Committee decided not to add Herceptin to the basket immediately but to reconsider it later (provided there is sufficient budget). With respect to choosing between Visudyne (t5) and left-ventricular assist devices (LVAD) (t6), it is easy to imagine decision-makers preferring LVAD (and adding it to the basket) as it has only marginally lower *Benefits* than Visudyne (t5) but is 21.6 million shekels cheaper. (It’s also worthwhile noting that LVAD was expected to save the lives of 12 patients facing imminent death, whereas Visudyne would reduce the risk of blindness for about 1000 people).

These first four additions to the basket (costing just 20.2 million shekels) would leave the Committee with the ‘abridged’ VfM Chart in Figure 3. Clearly, the new Pareto frontier – consisting of contraceptives (t16), Fuzeon (t14), Statins (t7), Visudyne (t5) and Herceptin (t3) – is closer to the diagonal than initially (Figure 2), which means that for these technologies the Committee is likely to find thinking about acceptable tradeoffs between *Benefits* and *Total Cost* (as always, subject to *Quality of Evidence* and *X-factors*) more challenging. The Committee could decide to add all of these technologies or just some of them – for example, the Committee would again be confronted with deciding whether or not to select Herceptin (t3) (still with the highest *Benefits* but also the highest *Total Cost* by a considerable margin). For the sake of keeping the exposition here simple, suppose that the Committee selected all five technologies on the new frontier (including Herceptin, and costing 172.9 million shekels in total). This decision would result in another Pareto frontier (not shown) – consisting of Humira (t18), long-acting insulins (t15), growth hormones (t11), Revlimid (t9), pain relief (t8). And so the prioritization process would continue, with the Committee performing its deliberations until the budget is exhausted.^f^

## Discussion

Most decision-makers charged with prioritizing health technologies, including Israel’s Basket Committee, already consider the four variables included in the VfM Chart, but, because of the complexity involved, typically not in such a systematic and transparent fashion. It is important to appreciate that our proposed framework is not intended to replace decision-makers’ value judgments in any way. On the contrary, the VfM Chart is intended to serve as a decision-support tool that is very much based on decision-makers’ value judgments.

This dependence on decision-makers’ value judgments can be appreciated by recognizing that, first of all, to construct the VfM Chart decision-makers’ must reveal their preferences about the relative importance of the dimensions comprising the points system for the incremental-benefits variable (as explained earlier, by answering the pairwise-ranking questions). In addition, decision-makers need to rate each technology according to its performance on the points system’s dimensions. Naturally, such rating exercises can be difficult because of the uncertainties involved, and so decision makers are likely to need to deliberate. For example, with reference to Table 2 again, should the impact of growth hormone on the HRQoL of short-statured children be rated as a ‘medium’ or ‘large’ gain? Is prolonging a cancer patient’s life by 5 months a ‘medium’, ‘small’ or perhaps even ‘large’ benefit? Moreover, such uncertainties are magnified by the criticism that can be easily directed at these performance levels (‘small’, ‘medium’ and ‘large’): that they are overly simplistic and not descriptive enough. For real-world applications the points system’s dimensions and levels would need to be refined for the prioritization exercise in hand.

Obviously, the total scores calculated for the incremental-benefits variable of the affected technologies are sensitive to how they are rated by decision-makers.^g^ Especially for new technologies, such uncertainties will almost always be compounded by deficiencies in the data available for forming judgments. Sensitivity analysis should be performed with respect to any controversial ratings to see what difference, if any, they make to the final decision about whether to add a technology to the basket or not. For each technology that looks like being rejected, and for which there is significant uncertainty surrounding any of its variables, decision-makers should ask themselves: “What would it take for this technology to be in contention (e.g. on or near the VfM Chart’s Pareto frontier), and how realistic is such a scenario?” The VfM Chart enables such ‘what-if’ experiments to be performed systematically.

The final respect in which the framework depends on decision-makers’ value judgments concerns the prioritization decisions themselves. As for all tools, how the VfM Chart is applied is at the discretion of decision-makers. They – rather than the tool – are ultimately responsible for deciding which technologies are selected. The VfM Chart simply displays the main variables for consideration and makes explicit the potential tradeoffs between the variables on the chart’s axes, where higher *Total Cost* can be compensated for by higher *Benefits*. It is up to decision-makers to determine the appropriate ‘rate of exchange’ between *Total Cost* and *Benefits* and also how to weigh the impact of *Quality of Evidence* and *X-factors,* all of which depend on value judgments.

The *X-factors* variable, in particular, serves as a potential ‘over-ride’ mechanism for enabling a particular technology to be prioritized ahead of others that are otherwise superior on the three other variables included in the VfM Chart. A well-known Israeli example is dental care for children (similar to t10 in Table 2 and Figures 2 and 3), which was introduced to the 2010 Basket Committee with a strict demand from the Deputy Health Minister, approved by the Cabinet, that it be added to the health basket, regardless any other considerations [40]. If decision-makers (or their political masters) choose to invoke such *X-factors*, they are, in effect, forced to explicitly explain why such a technology – with high *Total Cost* and/or low *Benefits* and/or poor *Quality of Evidence* relative to other technologies – ought to be added to the health basket in preference to others. The VfM Chart ensures such decisions are transparent (and auditable).

As mentioned in the Methods section, points systems have been widely used for diagnostic and treatment-based decision-making and for prioritizing patients for specific elective services. Somewhat surprisingly, points systems have not been so widely used for prioritizing technologies, though there appears to be increasing interest in doing so (e.g. see the references in [41]), including, for example, a recent report that argued for their greater use in the NHS [15]. One possible reason for this may be because, unlike diagnosing or prioritizing patients, prioritizing health technologies involves cost comparisons across technologies. Our proposed framework deals with this issue by including in the points system only dimensions related to the technologies’ incremental benefits, and then introducing their incremental costs to the prioritization exercise later when the VfM Chart is created.^h^

By focusing on each technology at the aggregate level – i.e. in terms of the effects of the overall intervention involving the technology on Israel’s population and health system respectively – the framework avoids the problems associated with using Incremental Cost-Effectiveness Ratios (ICERs) to prioritize technologies. Allocating a budget across possible interventions in reverse order of the technologies’ costs per QALY results in the maximization of QALYs only if two conditions are satisfied: (1) that interventions are sufficiently divisible for the technologies to be purchased in incremental units, and (2) that interventions are subject to constant returns to scale (so that changing how much of a technology is used affects the resulting health benefits by the same proportion) [42]. These two conditions seldom hold [43] – in which case, ICERs convey nothing about how affordable interventions are. Affordability is important information when allocating a budget; for example, a technology with a very low cost per QALY might be used to treat such a large number of people that its *total* cost is unaffordable (e.g. potentially in excess of the budget). Stephen Birch and Amiram Gafni recommend an alternative conceptual approach to using ICERs based on “determin[ing] whether in choosing to use some of [the available budget] for one particular intervention, the health gains produced by this intervention exceed the health gains that are foregone by not using the same resources for all other possible interventions.” (p. 49) [44]. “Because this involves the direct consideration of opportunity costs, measured in terms of health benefits foregone, it takes the form of a (non-monetary) cost-benefit analysis.” (p. 2099) [45]. The VfM Chart is consistent with this conceptual approach.

As well as using the VfM Chart to represent potentially any number of technologies under consideration at a point of time (e.g. when the Basket Committee meets annually), technologies from the past (funded and/or not funded) could be super-imposed for comparison purposes. The VfM Chart could also be used in a ‘dynamic’ manner consistent with Program Budgeting and Marginal Analysis [46]: as new technologies arise they could be introduced to the VfM Chart and considered for funding while, at the same time, old technologies are identified for decommissioning. Such a longitudinal focus would assist with achieving greater decision-making consistency over time.

Our proposed framework is compatible with the prioritization process currently followed by the Israeli Basket Committee, as summarized at the beginning of the article. In more detail here, this process begins with a discussion about each individual technology on its own – specifically, its contribution to patients’ health and society overall, independent of its cost. Technologies deemed deserving of further consideration proceed to the next stage where, after including cost data from the ‘Technical Sub-committee’, they are compared, subject to the budget constraint [8]. This prioritization stage comprises two rounds: in the first round, technologies that are judged not to be worthwhile given the budget constraint are discarded; and in the second round, the Committee compares the remaining technologies in order to choose those that should be added to the health basket and that can be afforded.

The approach used to make the final prioritization decisions over the last few years is that each Committee member nominates his or her ‘top ten’ technologies. Technologies nominated by a majority of members are written on the board in the meeting room. Other technologies nominated by fewer members are also written on the board and flagged with a question mark (indicating less support). The costs of all the technologies on the board are summed. If the total exceeds the budget, then in theory all technologies on the board are included for discussion with respect to being dropped until the budget is met; but in practice usually only the question-marked technologies are considered. The order in which technologies are discussed by the Committee can be critical, as the inclusion of one technology, given the budget constraint, necessarily means that one or more later candidates will be excluded.

We believe that the VfM Chart would be a useful decision-support tool at both rounds of the prioritization stage outlined above, especially the second round.^i^ All technologies that make it through to the prioritization stage could be represented in the VfM Chart, which could serve as the focal point to the Committee’s deliberations. In addition, the VfM Chart could be used as a powerful communication device to explain to stakeholders, including the general public, in an obvious visual fashion why particular technologies were prioritized over others. Such explanations might reduce feelings of injustice suffered by patients whose required technologies were not added to the health basket – to the extent, potentially, that even law suits might be averted.^j^

## Conclusion

The Value for Money Chart introduced in this article is an intuitively appealing decision-support tool for helping decision-makers to focus on the inherent tradeoffs involved in health technology prioritization. Such deliberations can be performed in a systematic and transparent fashion that can also be easily communicated to stakeholders, including the general public. The VfM Chart could be used by agencies like the Israeli Basket Committee that have to perform the ‘super-human’ mission of deciding which technologies to fund – a mission that must be performed each year, within a short period of time and under conditions of intense public interest and pressure.

The framework introduced here has not yet been applied in a real-world health technology prioritization exercise, but it is intended to be. An obvious area for future research is pilot-testing the VfM Chart using real data, thereby testing the framework’s usefulness. Ideally, this would involve working with the Basket Committee – including refining the points system for technologies’ incremental benefits to accurately reflect the preferences of Committee members and/or their constituencies. Likewise, the framework could be tested and applied by health technology prioritization agencies in other countries.

## Endnotes

^a^In addition, specific technologies could be similarly flagged if decision-makers have concerns about the reliability of the technologies’ cost estimates (e.g. inflated or, alternatively, unrealistically low).

^b^The sample comprised 61 Israelis – specifically, 44 professionals or researchers in healthcare or related fields (including 10 physicians and 7 health journalists), 5 representatives of patients’ organizations, and 12 members of the general public – plus 13 researchers from the Joint Center of Bioethics in Toronto, Canada [9].

^c^The 1000Minds software can be used to survey the preferences of large numbers of people; and so, if appropriate, the preferences of Israeli patients and taxpayers could be captured.

^d^Since then the data for several of the technologies – ones re-submitted to the Committee – have changed. The illustrative technology ‘dental care’ (for children) is based on data presented to the 2010 Basket Committee as well as media reports [40].

^e^Although, in theory, this optimization problem is similar to the classic ‘0-1 Knapsack Problem’ in Operations Research [47], it cannot be solved analytically using dynamic programming because of the need, potentially, to recognize *X-factors* – of *a priori* uncertain importance – on a technology-by-technology basis, in addition to the three other variables.

^f^The prioritization and budget-allocation process outlined here can be supported by the 1000Minds software mentioned earlier, which continuously keeps track of the total costs of the selected and unselected technologies as well as the remaining (unallocated) budget.

^g^The sensitivity of technologies’ total scores to measurement issues can be alleviated, at least in part, by introducing ‘mid’ levels with interpolated point values (i.e. between the main levels).

^h^In our earlier study [9], we experimented with including the *Total Cost* variable as a dimension in the points system via a survey analogous to the pairwise-ranking exercise explained earlier in the present article. Feedback from respondents revealed that this rendered the pairwise-ranking questions highly ambiguous (for an explanation, see section 4.3 of [9]). We concluded, therefore, that it is better to recognize *Total Cost* and *Benefits* as separate variables (as is usual in Cost-Benefit Analysis in general). Likewise, not including *Quality of Evidence* and *X-factors* in the points system used to construct the *Benefits* variable is justified by the likelihood that their relative importance to decision-makers is idiosyncratic to the particular technology considered (unlike health-related benefits, which are more generic).

^i^Some decision-makers suggested in personal communications with the authors that the VfM Chart would also be helpful for the health technology assessments performed by the Health Technologies Forum.

^j^For example, a petition brought to The High Court of Justice by multiple myeloma patients against the Minister of Health and others (including the Basket Committee) challenged the 2009 Basket Committee not to add the drug Revlimid for multiple myeloma (similar to t9 in Table 2 and Figures 2 and 3) [48]. The petitioners claimed they were discriminated against relative to other patients, especially people suffering from over-active bladders for which the Committee added a technology (similar to t13 in Table 2 and Figures 2 and 3) to the health basket. The petition was denied by the High Court of Justice which found that the Committee had not violated the law and nor had there been a failure to meet the standard of reasonableness in the Committee’s considerations and decisions. In our opinion, given that the Committee’s decision was justified, had the technologies under consideration been displayed on the VfM Chart, the decision and its justification, as well as the need to choose between Revlimid and medication for over-active bladder (if this pairwise choice had actually been necessary) would have been clearer and more understandable to stakeholders.

## Abbreviations

VfM: Value for Money; HRQoL: Health-related quality-of-life; QALY: Quality-Adjusted Life Year; PAPRIKA: Potentially All Pairwise RanKings of all possible Alternatives; NHS: National Health Service; ICERs: Incremental Cost-Effective Ratios.

## Competing interests

The first author (OG) declares she has no conflicts of interest. The second author (PH) owns the 1000Minds software referred to in the article, which he co-invented with Franz Ombler. Via their company 1000Minds Ltd, PH and FO earn income from software licenses, but they also make the software available for free to unfunded academic users (to-date >180 researchers and students worldwide).

## Authors’ contributions

The authors conceived of the article and wrote it together. Both authors read and approved the final manuscript.

## Authors’ information

Both authors have extensive experience and expertise in various aspects of prioritization decision-making; OG is a bioethicist and PH is a health economist. They started working together to develop the framework outlined in the article in 2008 when PH spent a sabbatical at The Gertner Institute for Epidemiology & Health Policy Research, where OG is a senior researcher.

## References

[B1] Rosen B Merkur S Israel: Health system review Health Syst Transit 2009 11 1 226 27050102

[B2] Yasour Beit-Or M The medications basket: the big winners – elderly and children [Hebrew] Ynet Health News 5 January 2011

[B3] Greenberg D Siebzehner MI Pliskin JS The process of updating the National List of Health Services in Israel: Is it legitimate? Is it fair? Int J Technol Assess 2009 25 255 261 10.1017/S026646230999016X 19619343

[B4] Tamir O Rabinovich M Shani M Year 2006 update of the Israel National List of Health Services Isr Med Assoc J 2006 8 595 600 17058406

[B5] Israeli A Chinitz D Updating the basket of health services [Hebrew] Harefuah 2003 142 100 102 159 12653041

[B6] Chinitz D Shalev C Galai N Israeli A The second phase of priority setting. Israel’s basic basket of health services: the importance of being explicitly implicit BMJ 1998 317 1005 1007 9841023

[B7] Shani S Yahalom Z The Israeli model for managing the national list of health services in an era of limited resources Law Policy 2002 24 133 147 10.1111/1467-9930.00131

[B8] Israeli Ministry of Health The Procedure of Updating the Health Services Basket – February 2010 2011 [Hebrew] Jerusalem [http://www.health.gov.il/hozer/sal_noal181010.pdf]

[B9] Golan O Hansen P Kaplan G Tal O Health technology prioritization: which criteria for prioritizing new technologies and what are their relative weights? Health Policy 2011 102 126 135 10.1016/j.healthpol.2010.10.012 21071107

[B10] Sabik LM Lie RK Priority setting in health care: lessons from the experiences of eight countries Int J Equity Health 2008 7 4 10.1186/1475-9276-7-4 18208617 PMC2248188

[B11] Nord E Balancing relevant criteria in allocating scarce life-saving interventions Am J Bioethics 2010 10 56 58 10.1080/15265161003633045 20379926

[B12] Kenny N Joffres C An ethical analysis of international health priority-setting Health Care Anal 2008 16 145 160 10.1007/s10728-007-0065-5 18449806

[B13] Holm S Goodbye to the simple solutions: the second stage of priority setting in health care BMJ 1998 317 1000 1007 10.1136/bmj.317.7164.1000 9841021

[B14] Hoedemaekers R Dekkers W Justice and solidarity in priority setting in health care Health Care Anal 2003 11 325 343 14769014 10.1023/B:HCAN.0000010061.71961.87

[B15] Devlin N Sussex J Incorporating Multiple Criteria in HTA. Methods and Processes. OHE Report 2011 London: Office of Health Economics

[B16] Stafinski T Menon D Philippon DJ McCabe C Health technology funding decision-making processes around the world: the same, yet different PharmacoEconomics 2011 29 475 495 10.2165/11586420-000000000-00000 21568357

[B17] National Institute for Health and Clinical Excellence Social Value Judgements. Principles for the Development of NICE Guidance 2008 London 27905706

[B18] Golan O Siegal G Health resources allocation – addition of new healthcare technologies to the Health Basket Israeli Bioethics and Health Lawforthcoming

[B19] Culyer AJ Bombard Y An equity framework for health technology assessments Med Decis Making 2012 32 428 441 10.1177/0272989X11426484 22065143

[B20] Hastie R Dawes RM Rational Choice in an Uncertain World. The Psychology of Judgment and Decision Making 2010 California: Sage Publications

[B21] Medal.org Limited Medal.org website [http://www.medicalalgorithms.com]

[B22] MacCormick AD Collecutt WG Parry BR Prioritizing patients for elective surgery: a systematic review ANZ J Surgery 2003 73 633 642 10.1046/j.1445-2197.2003.02605.x 12887536

[B23] Persad G Wertheimer A Emanuel EJ Principles for allocation of scarce medical interventions Lancet 2009 373 423 431 10.1016/S0140-6736(09)60137-9 19186274

[B24] Atkins D Best D Briss PA Eccles M Falck-Ytter Y Flottorp S Guyatt GH Harbour RT Haugh MC Henry D Hill S Jaeschke R Leng G Liberati A Magrini N Mason J Middleton P Mrukowicz J O’Connell D Oxman AD Phillips B Schünemann HJ Edejer TT Varonen H Vist GE Williams JW Jr Zaza S for the GRADE Working Group: Grading quality of evidence and strength of recommendations BMJ 2004 328 1490 1494 15205295 10.1136/bmj.328.7454.1490 PMC428525

[B25] Harris RP Helfand M Woolf SH Lohr KN Mulrow CD Teutsch SM Atkins D for the Methods Work Group U.S. Preventive Services Task Force Current methods of the US Preventive Services Task Force: a review of the process Am J Prev Med 2001 20 21 35 11306229 10.1016/s0749-3797(01)00261-6

[B26] Treadwell JR Tregear SJ Reston JT Turkelson CM A system for rating the stability and strength of medical evidence BMC Med Res Methodol 2006 6 52 10.1186/1471-2288-6-52 17052350 PMC1624842

[B27] Akers J Aguiar-Ibáñez R Baba-Akbari Sari A Beynon S Booth A Burch J Chambers D Craig D Dalton J Duffy S Eastwood A Fayter D Fonseca T Fox D Glanville J Golder S Hempel S Light K McDaid C Norman G Pierce C Phillips B Rice S Rithalia A Rodgers M Sharp F Sowden A Stewart L Stock C Trowman R Wade R Westwood M Wilson P Woolacott N Worthy G Wright K Systematic Reviews: CRD’s Guidance for Undertaking Reviews in Health Care 2009 York, UK: NHS Centre for Reviews and Dissemination, University of York

[B28] Belton V Stewart TJ Multiple Criteria Decision Analysis: An Integrated Approach 2002 Boston: Kluwer

[B29] Buckshaw D Decision analysis software survey. OR/MS Today 2010 37 http://www.orms-today.org/surveys/das/das.html

[B30] McFadden D Zarembka P Conditional logit analysis of qualitative choice behavior Frontiers in Econometrics 1973 New York: Academic Press

[B31] Ryan M, Gerard K, Amaya-Amaya M Using Discrete Choice Experiments to Value Health and Health Care 2008 Amsterdam: Springer

[B32] Drummond MF Sculpher MJ Torrance GW O’Brien BJ Stoddart GL Methods for the Economic Evaluation of Health Care Programmes 2005 Oxford: Oxford University Press

[B33] Hansen P Ombler F A new method for scoring multi-attribute value models using pairwise rankings of alternatives J Multi-Crit Decis Anal 2008 15 87 107 10.1002/mcda.428

[B34] 1000Minds softwarehttp://www.1000minds.com

[B35] Taylor W Laking G Value for money – recasting the problem in terms of dynamic access prioritisation Disabil Rehabil 2010 32 1020 1027 10.3109/09638281003775535 20380596

[B36] Hansen P Hendry A Naden R Ombler F Stewart R A new process for creating points systems for prioritising patients for elective health services Clin Govern Int J 2012 17 200 209 10.1108/14777271211251318

[B37] Fitzgerald A De Coster C McMillan S Naden R Armstrong F Barber A Cunning L Conner-Spady B Hawker G Lacaille D Lane C Mosher D Rankin J Sholter D Noseworthy T Relative urgency for referral from primary care to rheumatologists: the priority referral score Arthrit Care Res 2010 63 231 239 10.1002/acr.20366 20890984

[B38] Neogi T Aletaha D Silman AJ Naden RL Felson DT Aggarwal R Bingham CO III Birnbaum NS Burmester GR Bykerk VP Cohen MD Combe B Costenbader KH Dougados M Emery P Ferraccioli G Hazes JMW Hobbs K Huizinga TWJ Kavanaugh A Kay J Khanna D Kvien TK Laing T Liao K Mease P Ménard HA Moreland LW Nair R Pincus T The 2010 American College of Rheumatology/European League Against Rheumatism classification criteria for rheumatoid arthritis: Phase 2 methodological report Arthritis Rheum 2010 62 2582 2591 10.1002/art.27580 20872596 PMC3077961

[B39] Taylor WJ Singh JA Saag KG Dalbeth D MacDonald PA Edwards NL Simon LS Stamp LK Neogi T Gaffo AL Khanna PP Becker MA Schumacher HR Jr Bringing it all together: a novel approach to the development of response criteria for chronic gout clinical trials J Rheumatol 2011 38 1467 1470 10.3899/jrheum.110274 21724718

[B40] Even D Israel to fund dental care for kids at expense of other treatments Haaretz 2009 http://www.haaretz.com/print-edition/news/israel-to-fund-dental-care-for-kids-at-expense-of-other-treatments-1.2140

[B41] Tony M Wagner M Khoury H Rindress D Papastavros T Oh P Goetghebeur MM Bridging health technology assessment (HTA) with multicriteria decision analyses (MCDA): field testing of the EVIDEM framework for coverage decisions by a public payer in Canada BMC Health Serv Res 2011 11 329 10.1186/1472-6963-11-329 22129247 PMC3248909

[B42] Weinstein M Zeckhauser R Critical ratios and efficient allocation J Public Econ 1973 2 147 157 10.1016/0047-2727(73)90002-9

[B43] Birch S Gafni A Cost effectiveness/utility analysis. Do current decision rules lead us to where we want to be? J Health Econ 1992 11 279 296 10.1016/0167-6296(92)90004-K 10122540

[B44] Birch S Gafni A The biggest bang for the buck or bigger bucks for the bang: the fallacy of the cost-effectiveness threshold J Health Serv Res Policy 2006 11 46 51 10.1258/135581906775094235 16378532

[B45] Birch S Gafni A Incremental cost-effectiveness ratios (ICERs): the silence of the lambda Soc Sci Med 2006 62 2091 2100 10.1016/j.socscimed.2005.10.023 16325975

[B46] Mitton C Donaldson C Priority Setting Toolkit: A Guide to the Use of Economics in Healthcare Decision Making 2004 London: BMJ Books

[B47] Freville A The multidimensional 0-1 knapsack problem: an overview Eur J Oper Res 2004 155 1 21 10.1016/S0377-2217(03)00274-1

[B48] HCJ 434/09 Davidov v 2009 The Minister of Health [Hebrew] http://www.nevo.co.il/psika_word/elyon/09004340-w09.doc

